# FERTILITY CARE IN LOW- AND MIDDLE-INCOME COUNTRIES: Public sector access to medically assisted reproduction in South Africa: a case study

**DOI:** 10.1530/RAF-24-0072

**Published:** 2025-07-10

**Authors:** Gerhardus Marthinus Boshoff, Willem Ombelet, Carin Huyser

**Affiliations:** ^1^ Department of Obstetrics and Gynaecology, University of Pretoria, Pretoria, South Africa; ^2^ Faculty of Medicine and Life Science, Hasselt University, Hasselt, Belgium; ^3^ The Walking Egg Non-Profit Organisation, Genk, Belgium

**Keywords:** access, assisted reproduction, developing countries, LMIC, MAR, patient demographics, treatment progression

## Abstract

**Abstract:**

In South Africa, approximately 10% of the calculated need for medically assisted reproduction is being met due to limited access and unequal availability of these services. To facilitate understanding of challenges associated with access to assisted reproduction, a retrospective case study spanning 6 years was performed at one public sector hospital in South Africa offering these services. Demographic profiles, including income, region of residency and access to medical insurance, of patients seeking assistance to become pregnant were investigated. Patients were categorised as those who underwent diagnostic investigations only vs those who returned for therapeutic procedures, and the difference in demographic profiles between the two groups was determined. This investigation showed that patients from the lower-income classification group, without medical insurance, tend to return for therapeutic procedures less often than those with a higher income and medical insurance, even though these low-income patients qualify for a therapeutic procedure subsidy. An inverse relationship existed where patient numbers decreased as their travel distance increased, but patients who were required to travel further for assisted reproductive therapy tended to return for these procedures more often than patients who resided closer to the medical facility. In conclusion, access to medically assisted reproduction facilities is critically undersupplied and limited in the region. In order to ease the travel distance of patients, alternative primary diagnostic routes with accessible clinics are needed. In addition, costs of therapeutic procedures in the public sector should be re-evaluated to be offered at affordable rates for marginalised patients.

**Lay summary:**

In South Africa, about 10% of patients who need assistance to become pregnant are being helped. To better understand this phenomenon, researchers considered information about patients from a public sector hospital in South Africa. This includes how much money the patients earned, how far they travelled to the hospital and whether they had medical insurance. The patients were grouped into those who requested initial investigations but never returned for treatments, and those who returned for medical treatment. The differences between these groups were then evaluated. The research showed that people with less money tend to abandon further treatment more often, or take longer to return, than those with more money. The conclusion drawn is that assisted reproductive therapy is too expensive and that more IVF clinics are needed, using cheaper and simpler procedures of the same quality.

## Introduction

Medically assisted reproduction (MAR) has a centre-stage role in addressing infertility and assisting individuals and couples to achieve their reproductive goals (Afferri *et al.* 2022, Seiz *et al.* 2023). The optimal coverage of assisted reproductive technology (ART) is estimated at 1,500 ART cycles per year per million individuals in any given population (ESHRE Capri Workshop Group 2001, European Society of Human Reproduction and Embryology 2023). However, in South Africa, the South African Registry for Assisted Reproductive Technologies (SARA) reported that only approximately 10 and 13% of this calculated need was being met in 2015 and 2019, respectively (SARA 2019, 2024). (These percentages were calculated according to 7,950 total ART cycles reported for a population of 55 million people, and 9,439 total ART cycles for a population of 59.4 million people, respectively for 2015 and 2019, reporting on the period that the current study is focused on (SARA 2019, 2024)). In addition, the 2015 report did not indicate how many of these ART cycles were utilised by foreign patients. The 2019 report stated that more than 1200 ART cycles were initiated for cross-border care, resulting in lower ART utilisation by South African citizens, as South Africa is known to be a global hub for reproductive tourism in Sub-Saharan Africa (SARA 2019, 2024, Pande 2020, Moll *et al.* 2022, Whittaker *et al.* 2024).

South Africa faces significant challenges in providing adequate MAR, resulting in limited access and unequal availability of these services (Dyer *et al.* 2020, Connolly *et al.* 2021). In South Africa, accessibility to MAR (i.e. ART, surgical procedures, hormonal treatment and other interventions) is among the highest-ranking factors explaining the dissimilarity between the demand for ART cycles (intra-uterine insemination or ovarian aspiration with subsequent embryo culture) and the number of cycles being performed (Ombelet & Goossens 2017, Adamson *et al.* 2018, Ombelet & Onofre 2019, Dyer *et al.* 2020). Accessibility primarily relates to whether ART services of acceptable quality are available to a person or couple, and their ability to access these services from an economic and sociocultural viewpoint (Adamson *et al.* 2018, Dyer *et al.* 2020).

The geographic availability of ART services effectively speaks towards the number and placement of ART centres available to a given population. Although participation in SARA is not mandatory, the data provided is believed to be representative of most of the country’s MAR provided, especially the 2019 report. The registry reports indicate an increase in reporting from 16 centres in 2015 to 19 centres in 2019, of a total of 23 known ART units in South Africa (SARA 2019, 2024). In the 2015 report, the SARA group indicated that only four of the 16 centres reported performing 500 ART cycles or more per year, but there is no indication whether any of these centres were in the public sector (SARA 2019). However, it is known that ART in the private sector is dominant in South Africa and that the public sector units are most likely not among those performing more than 500 ART cycles per year (Connolly *et al.* 2021, Whittaker *et al.* 2024). Only three participants in the national ART registry publications are from public health sector academic hospitals, situated in two of the country’s nine provinces, with one in the Steve Biko Academic Hospital (SBAH) in the Gauteng province, and the other two in the Groote Schuur and Tygerberg Hospitals, respectively, both in the Western Cape province (SARA 2019, Government of South-Africa 2024). The SBAH unit is located north of the central region of the country, while the other two ART public service providers are situated close to the southernmost point of the country, approximately 1,450 km from SBAH, as calculated using Google Maps (Huyser & Boyd 2013). Patients from any of the other seven provinces in South Africa are required to travel through at least one provincial border to access ART services in the public sector (Government of South-Africa 2024). In a country of 1,219,813 km^2^, domestic travel implicates substantial distances to cover (Government of South-Africa 2024). Travelling from any of the other provinces ranges from 265 to 1,743 km to reach a public sector ART unit (Government of South-Africa 2024).

Apart from the well-documented lack of ART centres in the public sector of South Africa, the affordability of reproductive health screening and subsequent ART procedures should also be considered. South African medical professionals working in the field of MAR agree that access to ART should be increased (Whittaker *et al.* 2024). In Sub-Saharan Africa, the cost of MAR is often named as one of the major obstacles to overcome (Adamson *et al.* 2018, Botha *et al.* 2018, Dyer *et al.* 2020, Connolly *et al.* 2021, Njagi *et al.* 2023, Whittaker *et al.* 2024). According to studies by Dyer and colleagues (2013 & 2017), the cost of *in vitro* fertilisation (IVF) at one of the public sector ART centres in South Africa represents a calculated catastrophic cost (an expense that is so much more than a household can afford that it threatens the household’s financial survival) towards out-of-pocket payment for IVF in 20% of patients in general, and this increases to 50% of patients when considering the poorest third of the population only (Dyer *et al.* 2013, 2017). This does not even take into account the cost of explorative procedures to determine the couples’ aetiologies.

Expenses for investigative and therapeutic procedures may differ considerably between the public and private sectors, with occasionally subsidised MAR treatments in the public sector for low-income patients who are South African citizens without medical insurance (Matsaseng 2016, Connolly *et al.* 2021). The measure and type of subsidisation for ART procedures in the public sector differ across provinces and are based on provincial budget allocations. In addition, national and provincial human resource management, i.e. moratorium on posts, salary adjustments, retention of skills and infrastructure, minimises patient intake artificially. ART subsidisation therefore fluctuates annually and is also impacted by the different levels of ART procedures offered by the four different public sector facilities. Patients are indirectly subsidised through the coverage of infrastructure costs and salaries by public sector facilities, which can account for as much as 29% of the procedural cost according to one publication (Matsaseng 2016). Patients are exempted from having to cover these hospital expenses, or there is a stratified hospital fee that takes the patients’ income into account (Matsaseng 2016, Gauteng Department of Health 2023, Karaga *et al.* 2023). Direct subsidisation to patients usually includes a co-payment by patients, but the amounts and manner of subsidy changes from facility to facility and between provinces (Matsaseng 2016, Dyer *et al.* 2017, Gauteng Department of Health 2023). In general, even though the proportions differ, the subsidisation of MAR in the South African public sector allows for some patients to pay less for the facility fee at the hospital, while having to cover most, if not all, of the medication cost and laboratory fees from their own out-of-pocket funds (Matsaseng 2016, Dyer *et al.* 2017, Gauteng Department of Health 2023). The medication and laboratory fees can contribute to a significant proportion of the total ART cost, with laboratory fees reported to contribute as much as 35–48% of the direct expenses related to ART, while medication accounts for another 28% (Huyser & Boyd 2013).

At the time of the case study, the policy in the Gauteng province was that subsidisation of hospital fees, including ART cost, was calculated according to the patient’s income. Patients are classified into one of four categories, namely full payment (PP/PH/PF/PM), subsidised (H1/H2/H3), receiving free services (H0) or exempted to pay any fees (HG), according to the Gauteng Department of Health ‘Patient Classification Policy Manual’ (Gauteng Department of Health 2023). A patient is required to supply documentation during the initial registration to declare their level of income, i.e. a payslip/bank statement or complete a declaration of income form, or, if unemployed, provide an affidavit to this effect. This information is used to assign them to the relevant subsidisation categories (and subcategories). Full-paying patients are those whose annual income is above a set threshold for the current year, or are externally funded, not South African (with some exceptions) or have private medical insurance. Subsidised patients are grouped from H1 to H3, with patients in these categories receiving an 80%, 80% & 70% (H1, H2 & H3, respectively) subsidisation of costs associated with primary health care services. Although H1 and H2 categorised patients are eligible for the same percentage of subsidisation, H1 patients are subsidised for a more comprehensive list of medical procedures than H2 patients, listed as tariff categories described in the Gauteng Department of Health’s ‘Uniform Patient Fee Schedule’. In addition, patients classed as H0 and HG are exempted from paying for primary health care services and include, but are not exclusive to, patients without medical insurance who receive pension or social grants, are pregnant, less than 6 years of age or fall within the other criteria described in the policy manual. Patients are required to provide updated information yearly to review their classification status (Gauteng Department of Health 2023). These subsidies can relate to a substantial, almost 80%, reduction in the clinical and laboratory procedural fees for transvaginal ovarian aspiration, embryo culture and transfer. However, the subsidies exclude the payment of medication for controlled ovarian hyperstimulation, a significant portion of the total cost of ART (Njagi *et al.* 2023). Therefore, even when subsidised, the out-of-pocket costs for patients to bridge the financial gap from diagnostic to ART procedural costs may still be beyond the means of many households (Connolly *et al.* 2021, Njagi *et al.* 2023). Sampling of a small number from a middle-to-lower income group of patients at a public sector ART centre in South Africa indicated that the average income of patients attending the centre for diagnostic purposes only is 38% less than those continuing to therapeutic ART procedures (Huyser & Boyd 2013).

Through the understanding of these challenges, policymakers and healthcare professionals can work towards developing strategies to improve access to MAR in South Africa (Whittaker *et al.* 2024). The impact of the cost of ART provision in the public sector, which evidently impedes access to therapeutic ART services, requires scrutiny. This is underlined by the opinions expressed by medical professionals in the field of MAR in South Africa, who all agree that better access to ART is needed (Whittaker *et al.* 2024). To achieve the impact of ART expenses on access to MAR, we performed a retrospective study spanning 6 years at one of the three public sector hospitals in South Africa offering MAR. These data represent a case study focused on a single hospital in South Africa. However, this facility is the only public sector hospital providing MAR services in the northern half of the country and is known to perform the most ART cycles of all the public sector hospitals in South Africa. This facility’s data can therefore account for more than half of the country’s reported public sector ART activities (Karaga *et al.* 2023) and the authors consider the outcomes to be representative of a substantial proportion of MAR in the public sector of South Africa.

The demographic profiles of patients who entered the public health sector seeking assistance to become pregnant were compared, including income class, region or province of residency and access to medical insurance. The patients were categorised as those who underwent diagnostic investigations only vs those who also returned for therapeutic procedures, to determine the difference in demographic profiles between the two groups.

## Materials and methods

Recent provision and usage of ART services in the public sector of South Africa were explored retrospectively, with all information extracted from patient files at the Reproductive Biology Laboratory, SBAH, Pretoria, Gauteng, South Africa. The University of Pretoria’s Faculty of Health Sciences Research Ethics Committee approved the project as part of a comprehensive investigation into ART provision to marginalised patients (REC 149/2021).

**Figure 1:**
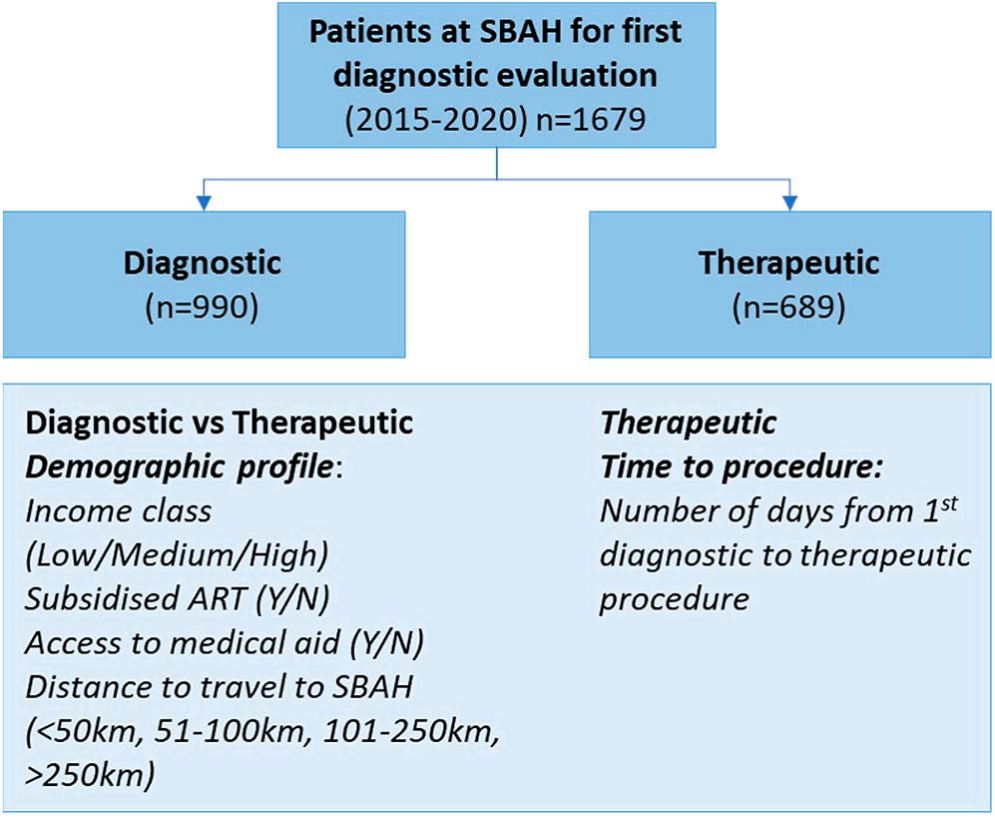
Allocation of patients to the diagnostic and therapeutic groups, based on the return for therapeutic procedures, with an indication of all demographic criteria evaluated.

The registration history of couples who initiated an investigation into their reproductive health status at SBAH was investigated (*n* = 1,679; first diagnostic visit 01 January 2015–31 December 2020). Demographic data (Fig. 1) were captured from clinical files, including i) income according to combined monthly income, then grouped per income class (low: €350, middle: €350–1,495, high: >€1,495), ii) qualification for subsidised care at SBAH, iii) access to medical insurance by either one of the partners and iv) the distance travelled to SBAH (<50 km, 51–100 km, 101–250 km, >250 km). Income groupings were calculated based on the study population’s quartile ranges according to the couple’s combined income as disclosed to the ART unit during their first visit. Private medical insurance classification was included as informative data supportive of the patients’ income classification. Payment for ART was not covered by any medical aid scheme in South Africa over the time period of this research study, as the first medical aid to provide this benefit launched it in January 2021 only (Discovery Health 2021). The selection of distance groupings was to include patients from within the same metropolitan area in the first category, patients from outside this area but from the same province in the second category, patients from adjacent provinces in the third and all others in the last category. In addition, the couples’ nationalities (South African or non-South African) were recorded for commentary purposes. However, since income and distance to travel were calculated from a quoted South African income and residing address, nationality was not considered when analysing demographic comparisons.

The couples’ initial diagnostic visit date and first therapeutic procedure date (if applicable) were captured, and the study population was grouped according to those with diagnostic evaluations only (not proceeding to therapeutic ART procedures; diagnostic group, *n* = 990) vs those that did return for therapeutic ART services (therapeutic group, *n* = 689). During data capturing, a 12-month cut-off time was considered sufficient to allow opportunity for patients to return for follow-up procedures, whereby patient recording stopped at the end of December 2020, and their return for therapeutic procedures was captured up to the end of December 2021. Within the therapeutic group, the time in number of days from initial diagnostic evaluation to first therapeutic procedure, termed time to procedure (TTP), was calculated. Using this information, demographic comparisons were made and discussed between various subpopulations.

The study population was split into two groups (diagnostic and therapeutic), for which one continuous variable (patient income), one ordinal variable (distance to travel) and two categorical variables (access to subsidy and medical insurance) were considered. Furthermore, the patients’ income groupings, which were calculated according to the study’s quartile ranges, were used for descriptive purposes and considered as an ordinal variable. Variables were compared using the Student’s 2-sample *t*-test and mean values with standard deviations reported, while the ordinal and categorical variables were evaluated using the Wilcoxon rank-sum and Fisher’s exact tests respectively, and data reported as frequencies and percentages. Nationality was noted as additional information. Nonetheless, most non-South African patients supplied a South African residential address, and the entire study population was considered as a group. Statistical analyses were performed by a biostatistician from the University of Pretoria (School of Medicine, Faculty of Health Sciences) using STATA 17 statistical software.

## Results

A summation of the parameters investigated for the entire study population can be seen in Table 1, with the overall average income reported, as well as the average income per classification group, in Euro. The study population’s average monthly income of €1,337.58 was observed to be similar to the 2018 South African average monthly income of €1,344.91 (Natalie 2023). All values reported in Euros are derived from South African Rand converted by ZAR16.016:€1 as per the average exchange rate from 2015 to 2020 (Nedbank Group Economic Unit 2024). Since the patient income groupings were calculated based on the study population’s quartile ranges, the average income of the three groups differed significantly (*P* < 0.001), as would be expected. A substantial difference was calculated between the low- and high-income groups’ average earnings, of which the former, at €209.26 per month, was a mere 7.2% of the latter’s €2,905.76 per month. The other parameters are reported as the total number of patients per subcategory, as well as the percentage breakdown of each category. Patients were predominantly South African, with 80% of couples indicating both partners as South African nationals.

**Table 1:** Summary of patient groups according to demographic categories. Data are presented as mean ± SD or as *n* (%).

Categories	Values
Income in euros	
All	1,337.58 ± 1,306.75
Low	209.26 ± 145.29
Middle	1,019.31 ± 455.06
High	2,905.76 ± 1,555.54
Income class	
Low	358 (21%)
Middle	884 (53%)
High	437 (26%)
Subsidised IVF	
Yes	797 (47%)
No	882 (53%)
Access to medical insurance	
Yes	706 (42%)
No	973 (58%)
Distance to travel	
<50 km	728 (43%)
51–100 km	564 (34%)
101–250 km	212 (13%)
>250 km	175 (10%)
Nationality	
Both RSA	1,348 (80%)
Male RSA, female foreign	43 (3%)
Male foreign, female RSA	57 (3%)
Both foreign	231 (14%)
Returned for therapeutic procedure	
Yes	689 (41%)
No	990 (59%)

**Figure 2:**
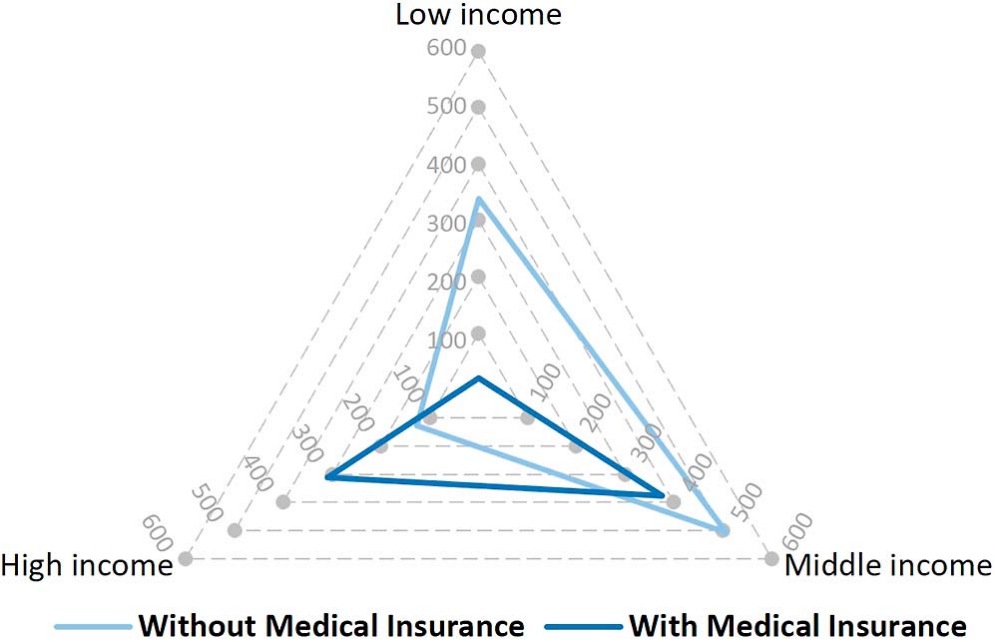
Radar chart (range 0–600) of patient numbers according to the triage of patients’ income classification (high, middle or low) vs their access to medical insurance.

**Figure 3:**
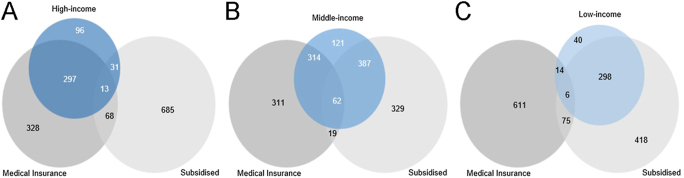
Venn diagrams of patient numbers showing the overlaps between access to medical insurance and subsidised ART procedures as well as (A) high-, (B) middle- and (C) low-income patients.

Higher patient income is known to provide access to medical insurance opportunities, where financial resources enable a person to afford higher insurance brackets (Discovery Health 2021). In addition, according to the subsidisation policies, this parameter is directly linked to the patients’ income level. The relationships were confirmed in the study population with Pearson’s correlation testing, indicating that there are significant medium relationships between income and access to medical insurance (positive relationship, *r* = 0.37, *P* < 0.001) and subsidisation (negative relationship, *r* = −0.47, *P* < 0.001). The same relationships were also observed when considering income according to the subgroupings, with 71 and 6% of patients in the high- and low-income groups having medical insurance, respectively. In Fig. 2, a radar chart depicts the distribution of patients with and without medical insurance as plotted over the three income categories. The two-dimensional chart displays the multivariate data on axes starting from the same mid-point. In the middle-income group, access to medical insurance was evenly distributed, with 57% of patients in this category presenting with medical insurance. A similar trend was observed in the subsidised IVF group classification (see Fig. 3), where 94.48% (753 of the 797) of eligible patients were from the low- and middle-income groups, while 93.88% (828 of the 882) of those who did not qualify for a subsidy were from the middle- and high-income groups. A significant difference (*P* < 0.001) was noted between the income groups when considering those who do not receive subsidised ART but have access to medical insurance (68%:36%:4% for high-, middle-, and low-income, respectively; Fig. 3). Due to the relationship between income and the two variables access to medical insurance and subsidisation being driven by income, further analyses used income as a single variate, with the assumption that the same relationships with income would be in place with access to medical insurance and subsidisation.

**Figure 4:**
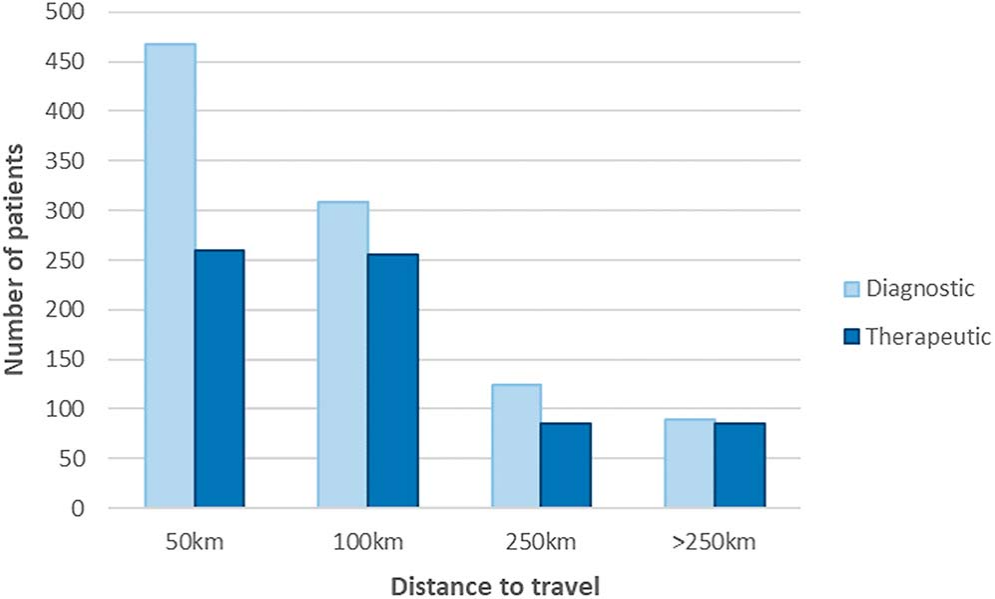
Graphical representation indicating a decrease in patient numbers as distance to travel increases, per subcategory diagnostic and therapeutic.

An inverse association was observed in the study population between the number of patients and travelling distance categories (*n* = 728 patients: <50 km; *n* = 564: 101–250 km; *n* = 212: 101–250 km; *n* = 175: >250 km). This was especially more noticeable in the diagnostic vs the therapeutic group, as shown in Fig. 4.

**Figure 5:**
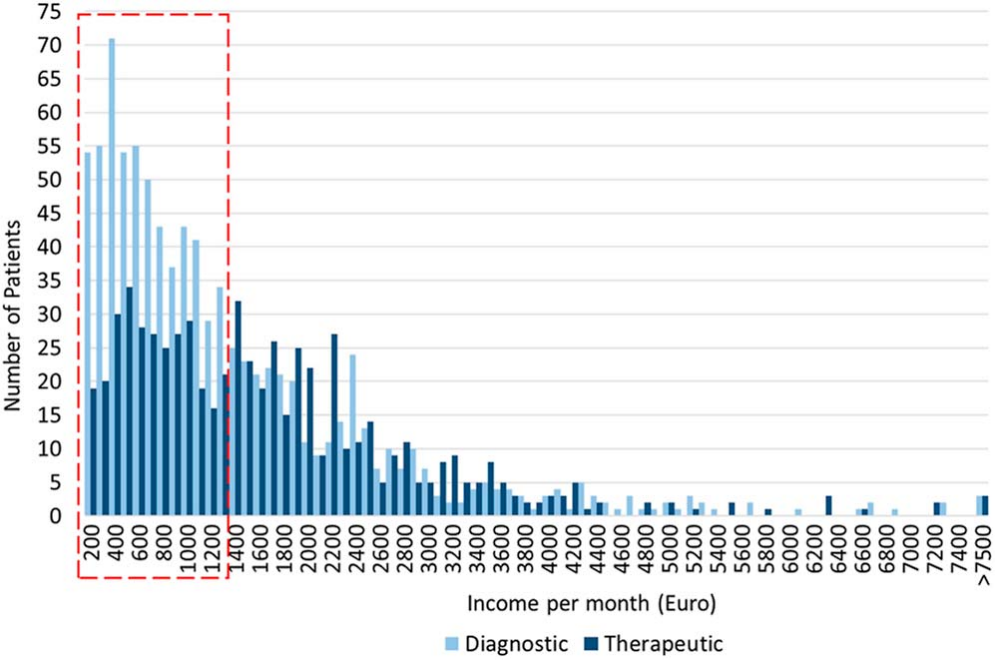
Number of patients in the diagnostic and therapeutic groups with income in €200 brackets. Patients below the population average indicated in a red block.

Comparing the diagnostic and therapeutic subpopulations, 689 couples (41% of the overall study population) returned for therapeutic procedures, as seen in Table 1, with a summary of the patients’ demographic distributions for the two groups seen in Table 2. A significant difference (*P* < 0.001) was seen in the return rate of patients who were or were not eligible for subsidised ART procedures (35 and 46%, respectively; Table 2). Similarly, patients from the low-income class who do not have medical insurance were less likely to return for therapeutic ART procedures than their high-income counterparts with medical insurance (30 vs 52%, *P* < 0.001; Table 2). The total number of patients in the therapeutic and diagnostic groups within specific income classifications, grouped according to the distance to travel, is indicated in Table 3. An income histogram (€200 brackets) for the diagnostic and therapeutic groups is displayed in Fig. 5, emphasising that patients within the diagnostic group are mostly earning below the study population’s average monthly income of €1,337.58. Patients who earn less than the study population’s average monthly income did not return for therapeutic procedures in 75% of cases, compared to 49% of cases when patients earn above the average (*P* < 0.001).

**Table 2:** Distribution of patients according to diagnostic and therapeutic groupings with reference to demographic categories income class, subsidisation, access to medical insurance and distance to travel.

	Diagnostic	Therapeutic
Income class		
Low	252	106
Middle	521	363
High	217	220
Subsidised IVF		
Yes	518	279
No	472	410
Access to medical insurance		
Yes	371	335
No	619	354
Distance to travel		
<50 km	467	261
51–100 km	309	255
101–250 km	125	87
>250 km	89	86

**Table 3:** Number of patients in the diagnostic and therapeutic groups, subdivided (i) per income group, (ii) distance to travel and (iii) an indication of the percentage diagnostic vs therapeutic patients per category.

	0–50 km		51–100 km		101–250 km		>250 km	
	Diagnostic	Therapeutic	Diagnostic	Therapeutic	Diagnostic	Therapeutic	Diagnostic	Therapeutic
Low income	138 (78%)	38 (22%)	79 (65%)	43 (35%)	23 (61%)	15 (39%)	12 (55%)	10 (45%)
Middle income	233 (65%)	127 (35%)	161 (53%)	140 (47%)	76 (62%)	47 (38%)	51 (51%)	49 (49%)
High income	96 (50%)	96 (50%)	69 (49%)	72 (51%)	26 (51%)	25 (49%)	26 (49%)	27 (51%)

The relationships between the patients’ likelihood to return for therapeutic procedures, their income and the distances that must be travelled were also considered. A small, significant positive relationship (*r* = 0.11, *P* < 0.001) was observed between income and the patients’ return for therapeutic procedures. In addition, a very small, significant positive relationship (*r* = 0.06, *P* = 0.013) was identified between the distance to travel and return for procedures. No significant relationship was observed between the patients’ income and the distance they travelled.

**Figure 6:**
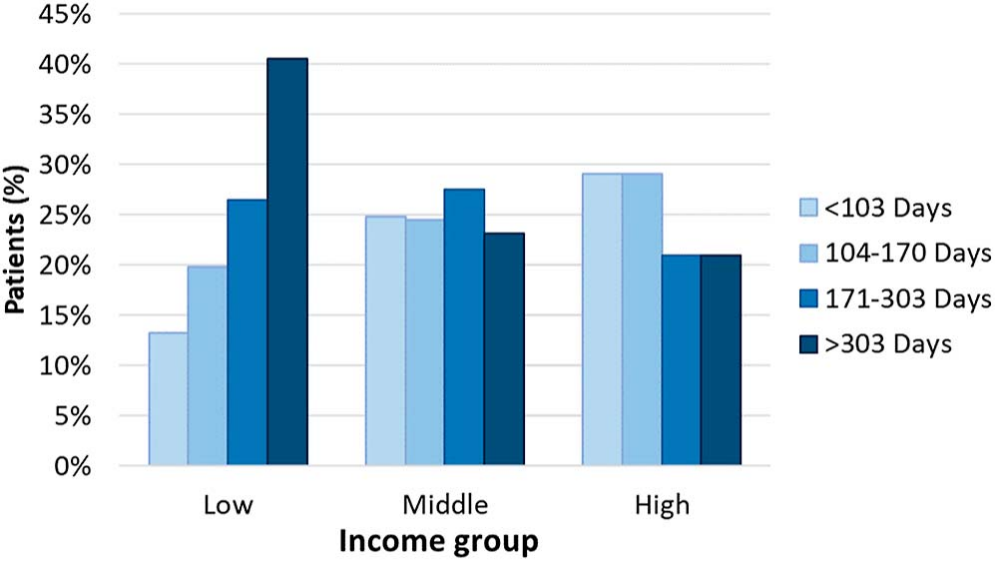
Therapeutic patients’ time to procedure according to four quartiles, per income group.

Within the therapeutic group, the average time to procedure (TTP) was 240 days from the patients’ first diagnostic evaluation to the first therapeutic procedure. With a median of 171 days, the distribution of patients’ TTP in four quartiles (<103, 104–170, 171–303, >303 days) is displayed per income group in Fig. 6 and per distance to travel category in Table 4. Patients from the low-income group returned for therapeutic procedures after more than the study population median of 171 days in 67% of cases, while only 42% from the high-income group took this long (*P* < 0.001). The relationships between TTP, income and distance to travel showed a small but significant negative relationship (*r* = 0.12, *P* = 0.002) between TTP and income, while there were no significant relationships between TTP and distance to travel or income and distance to travel.

**Table 4:** Number (%) of patients returning for therapeutic ART services per time to procedure quartile (days) plotted over the distance travelled, with an indication of the percentile distribution per distance category.

Time to procedure	0–50 km	51–100 km	10–250 km	>250 km
<103 days	53 (20%)	70 (27%)	22 (25%)	29 (34%)
104–170 days	71 (27%)	64 (25%)	22 (25%)	11 (13%)
171–303 days	65 (25%)	60 (24%)	22 (25%)	28 (33%)
>303 days	72 (28%)	61 (24%)	21 (24%)	18 (21%)

## Discussion

Data from the current case study confirmed that a large disparity exists in earnings of patients frequenting a public sector ART unit. The inequality in the income of the low-income group, earning only 7% of the wages of the high-income group, showcased the severity of the situation. The patients’ access to medical insurance and eligibility for subsidised ART procedures is linked to their monthly income, with the high- and low-income groups being representative of the patients with and without medical insurance, as well as not being subsidised and being subsidised respectively. The data indicated that patients in the low- income group, who are eligible for subsidisation and do not have access to medical insurance, have an approximately 70% drop-out rate to proceed from diagnostic to therapeutic procedures. In comparison, patients from the high-income group, who do have medical aid and are not eligible for subsidisation, return for therapeutic procedures in more than 50% of cases. Considering this difference in the number of patients returning for therapeutic procedures, a compelling argument can be made that the lack of funds and the cost of procedures have a direct impact and influence on patients’ decisions. The failure to return for therapeutic procedures is not necessarily an abandonment of the patients’ fertility journey. Some of the non-returns might be due to patients transferring to another fertility unit, either in the public or private sector, or deciding to adopt a child (Wiersema *et al.* 2006, Maxwell *et al.* 2018, Weis 2021). With patients who earn less than the study population’s average monthly income not returning for therapeutic procedures more often than patients who earn above average, the cost of procedures might be a factor when patients decide to return for MAR therapy. This is especially concerning when considered that 61% of patients earn below the study population’s average of €1,337.58.

Apart from being less likely to return for therapeutic procedures, low-income patients who do return for therapeutic procedures tend to progress slower to MAR therapy than their high-income counterparts. This can be witnessed via the incidence of a significantly higher percentage of patients from the low-income group returning for therapeutic procedures after more than the study population median time than from the high-income group. One might consider that patients from the low-income group require more time to budget and obtain funds to afford the necessary treatment, which is oftentimes an expense they cannot recover from (Dyer *et al.* 2017).

When considering the sensitive nature of MAR, a female’s optimal period to conceive is directly and inversely linked to her age (Liao *et al.* 2019, Pantos *et al.* 2020, Yang *et al.* 2023). According to Pantos *et al.* (2020), a single year’s difference in age relates to a statistically significant decrease in positive hCG and clinical pregnancy rates in patients 35 or 36 years old. With the study population’s average time between therapeutic procedures already being almost 6 months long, any delay causing a patient to wait longer than this to return for a therapeutic procedure, as is seen in more than half the cases in the low-income group, could have a negative impact on the patient’s probability to achieve a pregnancy. In addition, according to Speksnijder *et al.* (2024), the cumulative ongoing pregnancy rate after IVF of patients from lower socioeconomic environments was lower than their higher socioeconomic counterparts. This finding was based on the cumulative ongoing pregnancies of their study group in the Netherlands using a neighbourhood socioeconomic status calculation. Applying these two detrimental factors, increasing age and reduced cumulative ongoing pregnancy rate, a low-income patient who takes more than a year or two to return for the needed therapeutic intervention would have a cumulative diminished probability of a successful outcome. The high cost of MAR has an impeding effect and causes low-income patients to take longer to fulfil their wishes to become parents, and in some cases, denying them from achieving this goal at all.

An interesting phenomenon is seen when considering the combination of the patient’s income class and distance that they must travel for MAR. Patients from the lower income groups and with the shortest distance to travel (<50 km) do not return for therapy more often than those with the greatest travel distance (>250 km), showing a prominent decreased therapeutic return rate in the group with the least distance to travel. The necessity of multiple visits to an ART centre during a single ART cycle, combined with the lack of geographic accessibility of ART units, implies additional cost for travel and accommodation. The build-up of expenditures may be a significant and possibly unsurpassable extra expense for these patients (Huyser & Boyd 2013, Karaga *et al.* 2023). The shift in patient numbers might be an indication that patients who are required to travel further are less likely to initiate any form of MAR investigations, while those who initiate a MAR journey are committed and budgeted for the necessary expenses required for therapeutic procedures. The significant distance decay in patient numbers again reiterates the fact that more ART centres are needed in close proximity to be reasonably accessible.

The data reflects upon and is a reality check for a public sector hospital in South Africa, but the loss or fallouts emphasise inequalities relevant for all areas where MAR is not readily available or subsidised. This includes most middle- to low-income countries, especially those in Africa (DeWeerdt 2020, Afferri *et al.* 2022, 2024, Archary *et al.* 2023, Njagi *et al.* 2023, Seiz *et al.* 2023). Therefore, this is a call for action for MAR to be made more accessible to patients from lower income groups. The need for access to MAR to be increased is uniformly agreed upon by South African medical professionals in the field of MAR, but the way to do so is not so easy to agree upon (Whittaker *et al.* 2024). Strategically, this could be achieved through i) increasing support by means of subsidised treatment cycles, ii) reducing the cost of MAR and iii) increasing the number of IVF centres and medical professionals trained in MAR to reduce travel distances and associated expenses (Ombelet & Onofre 2019, Chiware *et al.* 2020, Afferri *et al.* 2024, Whittaker *et al.* 2024).

Some of these barriers might be overcome by the use of telemedicine, which has improved dramatically due to the Covid-pandemic, while keeping to high practice standard of care (Townsend *et al.* 2020, Prinsen 2023, Tran *et al.* 2024), or through basic investigative diagnostic procedures at remote clinics. Access to ART can be promoted through simplified diagnostic and ART procedures (Ombelet *et al.* 2025). For one such method, embryos cultured in the Walking Egg simplified IVF culture system have been reported to perform the same, and better, in the lab than sibling embryos resulting from ICSI and conventional culture when comparting fertilisation rate, with similar ongoing pregnancy, implantation and miscarriage rates after embryo transfer (Ombelet *et al.* 2022*b*). This embryo culture system is described to be effective for most patients in need of ART, only excluding those with moderate to severe male factor infertility or known fertilisation failure after IVF (Van Blerkom *et al.* 2014, Ombelet *et al.* 2025). The perinatal outcomes of babies born from this system were also compared to babies resulting from conventional culture in the same laboratory in Belgium (Ombelet *et al.* 2022*a*), singleton babies born from IVF/ICSI cycles in Belgium (Ombelet *et al.* 2023*b*) and all singleton babies born in Flanders, Belgium (Ombelet *et al.* 2023*a*). On all accounts the preterm birth and low birth weight rates were found to be favouring the simplified culture system, with similar (Ombelet *et al.* 2023*a*), if not better, results reported (Ombelet *et al.* 2023*a*,*b*). In addition, mild ovarian hyperstimulation has been shown to be an effective treatment option for good prognosis patients (Gianaroli *et al.* 2022, Nargund *et al.* 2022). Combining a low-cost simplified IVF culture system with mild ovarian stimulation could dramatically reduce the cost to patients for MAR, making MAR even more accessible to a large proportion of patients (Nargund *et al.* 2022, Ombelet *et al.* 2025).

Combining these strategies with the implementation of mobile ART laboratories can provide patients services at multiple medical facilities without the financial cost of setting up a laboratory at each facility (Büsing *et al.* 2021, Kroes *et al.* 2019, Kushnir *et al.* 2022,Ombelet *et al.* 2025). The reduced setup cost for a simplified IVF culture laboratory, further decreased through the shared costs by multiple facilities, would favourably lower patient fees. The use of a mobile ART laboratory is an academic exercise at this point (Ombelet *et al.* 2025). For the use of this type of laboratory to be implemented, a feasibility study would have to be performed, to establish the practicality of this plan. This would have to consider the necessary infrastructure requirements, based on the type of laboratory, availability of disposable items and consumables, and cost effectiveness to set up and operate this vehicle with sufficiently trained staff. The deployment and type of mobile laboratory should be carefully considered, to take into account the availability and willingness of trained medical professionals to work independently but within a team in such a mobile laboratory environment. When sufficient qualified health professionals are not available, which might well be the case in LIMC, training facilities should be established or current training programmes expanded to include the use of mobile laboratories in their repertoire. Experienced independent and self-employed consultants could also upskill to manage a mobile ART laboratory and be available to assist in the batching of patients on a regular basis. Furthermore, regulatory differences between countries and areas necessitate knowledge of the local legal requirements before the placement and operation of mobile laboratories.

In South Africa, a National Health Insurance (NHI) Act was accepted in 2023 and the Bill signed into law by the President of the Republic on the 15th of May 2024 (RSA Parliament 2023, National Department of Health 2024, RSA Government 2024). After signing the NHI Bill into law, the inevitable health system reform that should follow such a major policy change provides the ideal environment to address inequities and disparities to infertility treatment (Whyle & Olivier 2024). The aim of the NHI Act is to provide South Africa with universal access to health care services, as described in the NHI White Paper and the Constitution of South Africa (RSA Parliament 2023, RSA Government 2024). The NHI Fund will be the main purchaser and payer of healthcare services, with a defined package of services covered. For treatments and medicine not covered by the NHI, private medical insurance companies may still provide complementary cover while patients without medical insurance will have to pay for these services out-of-pocket (RSA Government 2024).

The NHI Act states that ‘in terms of section 27(1)(a) of the Constitution everyone has the right to have access to health care services, including reproductive health care’ (RSA Government 2024). While this has the potential to improve access to reproductive health services, infertility treatments are not explicitly listed as a covered service and the extent to which infertility treatments will be covered remains unclear (RSA Government 2024). Historically in South Africa, the focus on sexual and reproductive health has often been more on family planning to avoid unwanted pregnancies and on abortion than to assist patients who require MAR (Cooper *et al.* 2016, Lince-Deroche *et al.* 2016, Lince-Deroche *et al.* 2019). The gathering of commentaries and current discussions after the signing of the NHI Bill is the ideal time for stakeholders to act and ensure that access to MAR, and the lack thereof, is noticed by policy makers while there is a chance to address this inequity. This should be expanded further, to address the international requirement to address the problem of MAR being inaccessible to all and overly expensive (Kroes *et al.* 2019, Kushnir *et al.* 2022, Wang 2022, The Lancet 2024). Subsidisation of MAR needs to be addressed at the level of policy makers for regions with limited resources and large income disparities (Botha *et al.* 2018, Chiware *et al.* 2020, Connolly *et al.* 2021, Njagi *et al.* 2023). Furthermore, to reduce the cost of MAR, both diagnostic and therapeutic procedures should be simplified and affordable. This is achievable through low-cost IVF processes with mild ovarian stimulation (Gianaroli *et al.* 2022, Nargund *et al.* 2022) in tandem with subsidised medication (Njagi *et al.* 2023), combined with simplified IVF systems (Christiaens 2018), telemedicine and the use of mobile IVF laboratories (Kroes *et al.* 2019, Kushnir *et al.* 2022).

## Declaration of interest

The following interests are relevant: the data presented are part of a larger study performed during the principal author’s PhD research. All the authors are associated with the Walking Egg non-profit organisation. W Ombelet is an Associate Editor of *Reproduction & Fertility* and was not involved in the review or editorial process for this paper, on which he is listed as an author.

## Funding

The lead author’s research is supported through Hasselt University’s BOF-Bilateral Scientific Cooperation grant (reference number BOF21BL16).

## Author contribution statement

The principal author contributed to all aspects of the research, including data gathering, processing and write-up. All the authors were involved in development of the research plan, conceptualising of information and write-up of the article. 
